# Investigating Salivary Extracellular Vesicles as Biomarkers for Alzheimer's Disease: ExosomeAD Study Design and Baseline Characteristics

**DOI:** 10.1002/alz70856_105183

**Published:** 2026-01-09

**Authors:** Victoria Sanborn, Jonathan D. Drake, Hannah Alaimo, Em Teixeira, Jenna R Pracht, Charles Denby, Mandy G Pereira, Sicheng Wen, Peter J. Quesenberry, Jill A. Kreiling, Lori A. Daiello

**Affiliations:** ^1^ Warren Alpert Medical School of Brown University, Providence, RI, USA; ^2^ Rhode Island Hospital Alzheimer's Disease and Memory Disorders Center, Providence, RI, USA; ^3^ Rhode Island Hospital, Providence, RI, USA; ^4^ Rhode Island Hospital, Division of Hematology/Oncology, Providence, RI, USA; ^5^ Brown University, Providence, RI, USA

## Abstract

**Background:**

Brain‐derived salivary extracellular vesicles (EVs) contain mRNA, miRNA, and protein species which have the potential to be used for molecular characterization of brain health. Prior analysis of salivary EV mRNA identified Alzheimer's disease (AD)‐ and inflammation‐related biomarkers that may be diagnostically useful in this regard, however EV analysis is still in its infancy. The primary objective of this study is to identify a novel biomarker signature for AD using salivary EVs.

**Method:**

ExosomeAD is a 60‐month longitudinal cohort study enrolling older adults with normal cognition (CN; *n* = 150) and mild cognitive impairment (MCI; *n* = 50) at the Rhode Island Hospital Alzheimer's Disease and Memory Disorders Center. Baseline evaluation includes neuropsychological testing, self‐report inventories (mood, subjective cognitive impairment, daily functioning), vital signs, and collection of saliva and blood samples. Salivary EVs are being analyzed for mRNA, miRNA, and protein composition and compared with plasma biomarkers of AD risk assessed by PrecivityAD (C2N Diagnostics) testing (plasma Aß42‐40 ratio, APOE proteotype, and the Amyloid Probability Score (APS)). Participants complete up to 4 annual follow up visits (cognitive testing, surveys, and saliva/blood sample collection).

**Result:**

Currently, 183 participants (CN=163; MCI=20) have been enrolled. Participants in both groups to‐date are predominately female (CN, *n* = 115 (71%); MCI, *n* = 13 (65%). On average, participants with MCI (M_age_=77.7, standard deviation (SD)=5.6) are older than the CN group (M_age_=72.1 (5.1)). The group mean Montreal Cognitive Assessment total score in the current sample is lower among those with MCI (M=20.1 (4.1)) vs CN (M=27 (2.2)). MCI participants are more likely to be APOE4 carriers (71.4% vs CN, 31.1%), and have higher median APS (MCI_APS_=81.5; CN_APS_=17). More than half of participants endorsed family history positive for dementia (CN=60%; MCI=65%).

**Conclusion:**

Salivary EVs contain important information about brain health and may be a useful biomarker for AD. However, AD‐specific signatures in EVs have not yet been characterized. If successful, the Exosome Study will be first to demonstrate that salivary EV RNA and protein can be used to detect AD and facilitate the development of an inexpensive and noninvasive screening method for use in specialty and primary care settings.

